# Death of a Son is Associated With Risk of Suicide Among Parous Women in Taiwan: A Nested Case-Control Study

**DOI:** 10.2188/jea.JE20120060

**Published:** 2012-11-05

**Authors:** Chih-Cheng Chen, Chien-Chun Kuo, Trong-Neng Wu, Chun-Yuh Yang

**Affiliations:** 1 Department of Pediatrics, Kaohsiung Chang-Gung Memorial Hospital and Chang-Gung University, College of Medicine, Kaohsiung, Taiwan; 2 Division of Nephrology, Department of Internal Medicine, Kaohsiung Chang-Gung Memorial Hospital and Chang-Gung University College of Medicine, Kaohsiung, Taiwan; 3 Department of Public Health, China Medical University, Taichung, Taiwan; 4 Department of Public Health, College of Health Sciences, Kaohsiung Medical University, Kaohsiung, Taiwan; 5 Division of Environmental Health and Occupational Medicine, National Health Research Institute, Miaoli, Taiwan

**Keywords:** suicide, mortality, parity

## Abstract

**Background:**

The impact of the sex of a deceased child on maternal suicide has not been studied. We examined whether the death of a child, especially a son, increased the risk of suicide among parous Taiwanese women.

**Methods:**

This matched case-control study was done within a cohort of 1 292 462 Taiwanese women who experienced a first and singleton childbirth between 1 January 1978 and 31 December 1987 and were followed up until 31 December 2008. From the cohort, 2701 suicide cases were identified and 2701 controls were randomly selected. Multiple logistic regression was used to estimate the risk of suicide associated with the death of a child.

**Results:**

The adjusted odds ratios (ORs) for suicide among mothers whose son had died were 2.60 (95% CI = 1.18–5.73), 2.58 (1.28–5.20), and 4.20 (0.79–22.45) for death of a son aged younger than 1 year, 1 to 17 years, and 18 years or older. The ORs for suicide associated with the death of a daughter were not statistically significant: the respective adjusted ORs were 1.86 (0.82–4.62), 1.38 (0.54–3.49), and 2.48 (0.40–15.51).

**Conclusions:**

The death of a child, especially a son, increased the risk of maternal suicide, which supports the notion that preference for a son is firmly rooted in traditional Chinese culture.

## INTRODUCTION

In Taiwan, suicide is the ninth leading cause of death among men and the twelfth among women. In 2010, the age-adjusted mortality rate from suicide was 18.8 per 100 000 for men and 8.8 for women.^1^ Among the many traumatic life events that influence suicide risk, the death of a child is one of the most stressful,^2^ even more so than the death of a spouse.^3^^,^^4^ Indeed, several studies have documented greater mental distress and psychiatric problems among parents who suffered the loss of a child.^5^^–^^10^ Surprisingly, research on the association between losing a child and parental suicide has been limited.^11^^,^^12^

In a nested case-control study in Denmark,^12^ Qin and Mortensen studied 4 longitudinal registers that included 18 611 suicides (12 111 men and 6500 women) of individuals aged 18 to 75 years from 1981 to 1997 and 372 220 matched control subjects. They found an increased risk of suicide after the death of a child. Li et al^11^ conducted a prospective study in Denmark that investigated 21 062 parents who lost a child (exposed cohort) and 293 745 controls (parents with no death of a child and whose family structure matched that of the exposed cohort). Their results showed that the death of a child was correlated with an increase in deaths from unnatural causes (including suicide and accidents).

Suicide and death of a child are rare events. The limited empirical evidence linking the effect of losing a child with parental suicide may be the result of deficiencies in sample size and study design. Only studies of representative suicides from the general population would have sufficient statistical power to detect an association between the 2 rare events of death of a child and suicide.^11^^,^^12^

The aforementioned studies that explored the impact of the death of a child on parental suicide were conducted in a country with an advanced economy (Denmark). However, the association between death of a child and parental suicide remains unexplored in more culturally traditional nations. In Chinese societies, male heirs are highly prized for economic reasons and for their ability to continue the family lineage. As a result, women with daughters will continue to bear children until they give birth to a son,^13^ which may be related to the Chinese preference for a son.^14^^,^^15^ A previous study found that parity was inversely correlated with the suicide rate among parous women in Taiwan.^16^ To our knowledge, no research has documented the impact of the sex of a deceased child on maternal suicide. Therefore, to assess whether the death of a child, and in particular a son, increased the risk of suicide among parous women, we conducted the present nested case-control study, using a cohort of Taiwanese women who first gave birth to a singleton child between 1978 and 1987.

## METHODS

### Data source

This study includes 2 population-based registries: Taiwan’s National Death Certification Registry and National Birth Registry. We linked records for each individual across the 2 registries using the resident’s registry number, which uniquely identifies all residents of Taiwan.

The National Death Certification Registry includes data on sex, year of birth, and the date and cause of death for all Taiwan residents. By law, a death certificate must be completed and registered within 1 month of a death. All death certificates are reviewed and coded in the central office of the National Death Certification Registry by registrars with medical training. Taiwan’s practices for cause-of-death coding are considered excellent and yield highly accurate data.^17^

The National Birth Registry was converted to electronic format starting in 1978 and includes information on all live-born children in Taiwan. Births in Taiwan must be registered at the local household registration office by the infant’s family within 15 days of the birth. The registration form is completed by the physician who attended the delivery and includes information on parental age, education, parity, gestational age, date of delivery, and infant sex and birth weight. The National Birth Registry data are regarded as complete, reliable, and accurate because most deliveries take place in a hospital or clinic^18^ and because the birth certificates are completed by the attending physicians. Furthermore, registration of all live births at local household registration offices is mandatory and is required to document the child’s citizenship.^19^^,^^20^

Because the dataset used in this study consists of secondary data released to the public for research purposes, the study was exempt from full review by an institutional review board.

### Identification of cases and controls

The study cohort consists of all female residents of Taiwanese nationality who, between 1 January 1978 and 31 December 1987, had a first and singleton live birth in Taiwan that was recorded in the National Birth Registry. Cohort entry date is therefore the date of birth of their first child, during the above period. Cohort members were followed up until death or 31 December 2008. We performed a nested case-control study to identify the impact of child loss on maternal suicide. A death in the cohort was classified as a suicide if linking the unique resident number to the National Death Certification Registry yielded a cause of death identified as suicide (International Classification of Diseases, Ninth revision, codes E950–E959). For each case of suicide, we randomly selected 1 control among the cohort (with the requirement that controls be alive and at risk for suicide at the time of matching). To avoid selection bias, we used density sampling (selection of an individual on a specific day), so that an individual could be selected as a control before she herself became a suicide case. Furthermore, a subject could serve as a control for different cases, ie, more than once.^21^ Control subjects were matched pairwise to cases by age (same year of birth), index date, duration of follow-up, and place of residence. We defined duration of follow-up as the length of time from the date of cohort entry to the index date.

### Ascertainment of information about children

We obtained information on the children of each case and control by linking maternal personal identification numbers to the National Birth Registry. By so doing, we identified children from cohort entry to index date (date of suicide death for cases and date of selection for controls). In turn, we ascertained the vital status of children by linking their personal identification numbers with the National Death Certification Registry to identify the date and cause of death of any children. Persons not linked to a death record were considered to be alive.

### Statistical analysis

We used the chi-square test for categorical variables to determine the statistical significance of differences in frequency distributions among cases and controls. Odds ratios (ORs; estimates of the relative risk of suicide) and 95% CIs were obtained using various multivariate conditional logistic regression models, including a model that accounted only for matching factors (age at index date, place of residence, duration of follow-up; basic model) and models that controlled for other potential confounders, such as marital status (married, unmarried [including widowed, divorced], and never married), duration of education (≤9, >9 years), approximate decade of cohort entry (1978–1990, 1991–2000, 2001–2008), maternal age at first childbirth (age at entry, ≤20, 21–24, ≥25 years), number of children (1, 2, 3, ≥4), and age of youngest child at suicide or index time. Two-sided tests were used for all models, and a *P* value of less than 0.05 was defined as statistically significant. All analyses were performed using the SAS statistical package (version 9.2, SAS Institute Inc).

## RESULTS

During the 33 723 528 person-years of follow-up of the study cohort (1 292 462 women followed for a maximum of 30.92 years and a median of 26.33 years), we identified 2703 suicide deaths. No controls could be found for 2 of these 2703 ascertained cases. A total of 2701 suicide deaths and 2701 controls were thus included in the analysis.

The characteristics of the 2701 suicide cases and 2701 matched controls are shown in Table 1. Cases and controls were well matched with regard to age at index date, place of residence, age at cohort entry, and duration of follow-up. Cases were more likely to be unmarried and were less educated. Cases were slightly more likely than controls to enter the cohort after 2000.

**Table 1.** Characteristics of the study population

Variable	Cases (*n* = 2701)	Controls (*n* = 2701)	Chi-square test *P* value
Age at index date, y			
<30	678 (25.1%)	678 (25.1%)	*P* = 0.99
30–39	728 (26.95%)	728 (26.95%)	
40–49	1040 (38.5%)	1043 (38.62%)	
>49	255 (9.44%)	252 (9.44%)	
Age at cohort entry, y			
≤20	788 (29.17%)	792 (29.32%)	*P* = 0.99
21–24	1185 (43.87%)	1181 (43.72%)	
≥25	728 (26.95%)	728 (26.95%)	
Year of cohort entry			
1978–1990	772 (28.58%)	822 (30.43%)	*P* = 0.03
1991–2000	1048 (38.80%)	1029 (38.10%)	
2001–2008	881 (32.62%)	850 (31.47%)	
Duration of follow-up, y			
Mean ± SD	15.04 ± 8.47	15.04 ± 8.47	—
Years of schooling			
≤9	1830 (67.75%)	1654 (61.24%)	*P* < 0.001
>9	871 (32.25%)	1047 (38.76%)	
Marital status			
Married	2593 (96%)	2634 (97.52%)	*P* = 0.002
Unmarried	108 (4%)	67 (2.48%)	
Place of residence			
Urban	1464 (54.2%)	1464 (54.2%)	—
Rural	1237 (45.8%)	1237 (45.8%)	

Table 2 shows summary statistics for cases and controls. As compared with controls, cases were more likely to have a child who had died (4.70% vs 3.18%) and less likely to have 3 or more children (33.58% vs 38.80%). There was no difference between groups in the proportion of mothers who had a young (<9 years) child (44.54% vs 44.87%).

**Table 2.** Information regarding children of cases and controls

	Cases (*n* = 2701)	Controls (*n* = 2701)	Chi-square text *P* value
No. of children^a^			
1	624 (23.10%)	500 (18.51%)	*P* < 0.001
2	1170 (43.32%)	1153 (42.69%)	
3	676 (25.03%)	776 (28.73%)	
4+	231 (8.55%)	272 (10.07%)	
Sex of deceased child			
No child death	2574 (95.30%)	2615 (96.82%)	*P* = 0.013
Female	50 (1.85%)	38 (1.41%)	
Male	77 (2.85%)	48 (1.77%)	
Age of deceased child, y			
No child death	2574 (95.30%)	2615 (96.82%)	*P* = 0.023
Death of a child			
<1	52 (1.93%)	38 (1.41%)	
1–17	60 (2.22%)	42 (1.55%)	
≥18	15 (0.55%)	6 (0.22%)	
Age of youngest child, y			
≥18	717 (26.55%)	692 (25.62%)	*P* = 0.88
10–17	860 (31.84%)	881 (32.62%)	
1–9	964 (35.69%)	968 (35.84%)	
<1	239 (8.85%)	244 (9.03%)	
Time since the death of a child, y			
No child death	2574 (95.30%)	2615 (96.82%)	*P* < 0.001
<1	31 (1.15%)	6 (0.22%)	
≥1	96 (3.55%)	80 (2.96%)	

^a^Including decedent.

Table 3 shows the associations between suicide and death of a child. The effect of a death of a child was less when the deceased was a daughter, and the ORs for suicide associated with loss of a daughter were not statistically significant. The OR for suicide associated with death of a son aged 1 to 17 years was 2.08 (95% CI = 1.07–4.03). In the multivariate-adjusted model (model 2), the ORs were 2.60 (95% CI = 1.18–5.73), 2.58 (1.28–5.20), and 4.20 (0.79–22.45) for death of a son aged younger than 1 year, 1 to 17 years, and 18 years or older, respectively. The OR for mothers whose son died at age 18 years or older was not statistically significant, but this may have been due to the small number of mothers in this group (*n* = 15 cases, *n* = 6 controls).

**Table 3.** Odds ratios (ORs) for suicide associated with the death of a child

	Model 1^a^	Model 2^b^	Model 3^c^
	OR (95% CI)	OR (95% CI)	OR (95% CI)
Age of deceased child, y			
No child death	1.00	1.00	
<1			
Female	1.54 (0.69–3.45)	1.86 (0.86–4.62)	
Male	2.03 (0.95–4.34)	2.60 (1.18–5.73)	
1–17			
Female	1.18 (0.48–2.94)	1.38 (0.54–3.49)	
Male	2.08 (1.07–4.03)	2.58 (1.28–5.20)	
>18			
Female	2.53 (0.43–14.91)	2.48 (0.40–15.51)	
Male	3.51 (0.68–18.27)	4.20 (0.79–22.45)	
Time since child death, y			
No child death	1.00		1.00
<1			
Female	2.33 (0.60–9.02)		2.29 (0.57–9.19)
Male	8.00 (1.84–34.79)		8.69 (1.95–38.71)
≥1			
Female	1.27 (0.68–2.37)		1.53 (0.81–2.91)
Male	1.63 (0.97–2.74)		2.11 (1.22–3.66)

^a^Model 1 was adjusted for age at index date, place of residence, and duration of follow-up.

^b^Model 2 was adjusted for the variables in model 1 plus marital status, years of schooling, age at first birth, year of cohort entry, number of children, age of youngest child, and age of deceased child.

^c^Model 3 was adjusted for the variables in model 1 plus marital status, years of schooling, age at first birth, year of cohort entry, number of children, age of youngest child, and time since the death of a child.

Last, we examined the timing of the death of a child in relation to maternal suicide. No association was observed for the death of a daughter, regardless of time since the daughter’s death. However, the ORs were statistically significant among women who had lost a son. The adjusted ORs associated with the death of a son within 1 year and more than 1 year before maternal suicide were 8.69 (95% CI = 1.95–38.71) and 2.11 (1.22–3.66), respectively.

## DISCUSSION

We found that the risk of maternal suicide increased after the death of a son younger than 18 years but did not significantly increase after the death of a daughter. In addition, the risk of maternal suicide was particularly high in the first year after the death of a son.

In Chinese societies, sons are highly prized because they are expected to care for their elderly parents.^13^ In most Asian countries with limited social welfare systems, daughters marry out of the family; therefore, sons must shoulder the responsibility of looking after their parents. In such patrilineal societies, sons also fulfill the culturally important role of carrying on the family name.^13^ Given these societal preferences for a son, we hypothesized that the death of a son might result in greater bereavement as compared with the death of a daughter. Our results confirm this hypothesis: the risk of maternal suicide increased to a greater extent after the loss of a son than after the loss of a daughter. To our knowledge, this study is the first to show that maternal suicide risk varies according to the sex of the deceased child. On the basis of the reversibility criterion,^22^ this finding is additional evidence that preference for a son is firmly rooted in traditional Chinese culture.

Our results showing an association between the death of a son younger than 18 years and increased maternal suicide risk are consistent with those of a study conducted in Denmark.^12^ The present and past findings indicate that parents who lost a child had more mental distress and psychiatric manifestations^7^^,^^8^^,^^10^; in particular, parents who experienced the death of a young child reported more grief, depression, and shock than did parents of older decedents.^23^

Grief is often most intense soon after the death of a child.^2^^,^^10^ The most extreme reactions are usually observed soon after the child’s death, and there is a short-term peak in the rate of deaths from unnatural causes during this period.^24^^–^^26^ Therefore, our finding of a greater risk of suicide during the first year after the death of a child is consistent with this conceptual framework of peak bereavement and grief. Few studies, however, have analyzed the relationship between the time elapsed since the loss of a child and maternal suicide risk.^12^ Overall, our findings conform with those of the aforementioned Danish study, although the present study also showed that maternal suicide risk varied according to the sex of the child, which likely reflects the cultural values of traditional Chinese societies.^12^ Our results strongly suggest that mothers who lose a son require familial and social support, particularly in the first year after a child's death.

Our study has several strengths. First, it is free from selection bias because we used a study cohort composed of a population-based database that uniquely identifies all suicides by means of a national identification number, supplemented with control subjects selected from a density sampling of the study cohort. Second, information on exposures and confounders was extracted from the aforementioned database, ie, all data were collected prospectively and independently of the research hypothesis. Thus, recall bias from exposure data is not a concern.

Our study does, however, have some limitations. First, because our dataset lacks information on social networks and activities, we were unable to control for the potential confounding effect of maternal participation in social organizations^27^ and religious activities,^28^ which may protect against suicide. Nevertheless, our population was less likely to engage in social organizations and religious activities than are populations in northern European countries (Norway, Denmark, and Finland), due to differences in cultural factors.^29^ The confounding effects of these 2 factors, if they exist, are likely to be small. Furthermore, adjustment for these 2 confounders would not qualitatively change the results if their effects with respect to maternal suicide were smaller than the effect of losing a son. It is not known if employment protects against suicide, because data on the occurrence, duration, and timing of unemployment are not always available. However, we believe that unemployment did not have a substantial effect on our results because it was rare in Taiwan during the study period.

Second, subject misclassification is a potential concern, although the manner in which death certificates are completed in Taiwan mitigates this possibility. In principle, death certificates must be completed by a physician. If the cause of death is uncertain, the police require a forensic medical examination, after which a formal finding is jointly determined by a prosecutor and coroner, both of whom are primarily concerned with the possibility of homicide.^30^ A determination of suicide helps to exclude this possibility. Nonetheless, it cannot be ruled out that some suicides were misclassified as accidental deaths. However, any such misclassification is unlikely to be related to the death of a male child and would only bias our results downward.

Third, women aged 65 years or older have the highest suicide rate in Taiwan.^31^ Because the present study population is relatively young in comparison (mean age at suicide, 38.2 ± 9.4 years), and had not reached the age associated with the highest suicide risk, the generalizability of our findings may be limited.

Fourth, we do not have information on the prevalence of major depression or complicated grief among mothers who experienced the death of a child. Although suicide reflects a psychiatric disorder or severe distress, the prevalence of major depression or complicated grief might be similar among mothers, regardless of the sex of the decedent. If so, the present results might not be due to the Chinese cultural preference for sons.

In summary, we found that the risk of maternal suicide increased after the death of child, particularly after the death of a son. This finding is additional evidence that preference for a son is firmly rooted in traditional Chinese culture.
